# The Impact of Thromboprophylaxis on the Survival of Patients with Advanced Pancreatic Cancer. The Pancreatic Cancer and Tinzaparin (PaCT) Study

**DOI:** 10.3390/cancers13122884

**Published:** 2021-06-09

**Authors:** Michalis V. Karamouzis, Ilias Athanasiadis, Georgios Samelis, Christos Vallilas, Alexandros Bokas, Adamantia Nikolaidi, Areti Dimitriadou, Panagiotis Sarantis, Nikolaos Pistamaltzian, Dimitrios Schizas, Alexandros Papalampros, Evangelos Felekouras, Dimitrios Dimitroulis, Eustathios Antoniou, Georgios Sotiropoulos, Pavlos Papakotoulas

**Affiliations:** 1 Molecular Oncology Unit, Department of Biological Chemistry, Medical School, National and Kapodistrian University of Athens, 11527 Athens, Greece; chris-vallilas@hotmail.com (C.V.); panayotissarantis@gmail.com (P.S.); 2 Oncology Department, Mitera Hospital, 15123 Marousi, Greece; iliasathanasiadis40@gmail.com (I.A.); mantonikolaidi@gmail.com (A.N.); nfpist@gmail.com (N.P.); 3 Oncology Unit, Hippokration General Hospital, 11527 Athens, Greece; oncologydept.samelis@hippocratio.gr (G.S.); dim.areti@hotmail.com (A.D.); 4 1st Clinical Oncology Department, Theagenio Cancer Hospital, 54639 Thessaloniki, Greece; alexanderbokas@outlook.com (A.B.); papakotoulas@gmail.com (P.P.); 5 First Department of Surgery, Medical School, National and Kapodistrian University of Athens, 11527 Athens, Greece; schizasad@gmail.com (D.S.); apapalampros@med.uoa.gr (A.P.); evangelosf@hotmail.com (E.F.); 6 Second Department of Surgery, Medical School, National and Kapodistrian University of Athens, 11527 Athens, Greece; dimdimitr@med.uoa.gr (D.D.); efstantoniou@med.uoa.gr (E.A.); geosotirop@med.uoa.gr (G.S.)

**Keywords:** pancreatic cancer, LMWHs, tinzaparin, survival, chemotherapy, thromboprophylaxis

## Abstract

**Simple Summary:**

Pancreatic cancer (PaC) induces a prothrombotic and hypercoagulable state. Thrombosis occurs in 20% of PaC patients and is associated with worse prognosis and reduced progression-free survival (PFS). The aim of this retrospective observational study (PaCT) was to investigate the effect of thromboprophylaxis with an intermediate dose of tinzaparin on the PFS of patients treated with nab-paclitaxel and gemcitabine. Data obtained from 110 patients with active PaC administered prophylaxis with tinzaparin during the study resulted in median PFS of 7.9 months; data for the PFS of patients without simultaneous anticoagulation were obtained bibliographically from 14 studies, and after applying meta-analysis was 5.6 months. Patients receiving anticoagulation with tinzaparin had 39.5% higher PFS than patients without such thromboprophylaxis (*p* < 0.05). During follow-up, three (2.7%) thrombotic events and two (1.9%) clinically relevant non-major bleeding events occurred. Concluding, PFS in advanced PaC patients undergoing chemotherapy was positively impacted by thromboprophylaxis with intermediate dose tinzaparin.

**Abstract:**

Pancreatic cancer (PaC) induces a prothrombotic and hypercoagulable state. The aim of this study was to investigate the effect of tinzaparin in combination with chemotherapy. The PaCT (pancreatic cancer and tinzaparin) study was a retrospective observational study that collected data regarding progression free survival (PFS) in advanced or metastatic PaC patients who received thromboprophylaxis with tinzaparin during chemotherapy with nab-paclitaxel (N) and gemcitabine (G). The primary end point was to compare, from already published data, the PFS of patients receiving thromboprophylaxis with tinzaparin with the PFS of patients receiving chemotherapy with N–G but no thromboprophylaxis. Secondary end points were efficacy and safety of anticoagulation. In total, 110 PaC patients, 93% with advanced or metastatic disease, treated with N–G and tinzaparin (10,291 ± 1176 Anti-Xa IU, OD, median duration 8.7, IQR: 5.6–11.9 months) were enrolled. Of these, 52% were males and; the median age was 68 (40–86 years). The tumor was located to in the pancreatic head at in 45% of the patients. The median PFS was 7.9 months (IQR: 5.0–11.8 months). Out of 14 similar studies (involving 2994 patients) identified via systematic search, it was determined that the weighted PFS of patients receiving N–G but no anticoagulation was 5.6 months. Therefore, patients receiving tinzaparin had 39.54% higher PFS than patients without thromboprophylaxis (*p* < 0.05). During the follow-up period of 18.3 ± 11.7 months, three (2.7%) thrombotic events were recorded while two clinically relevant non-major bleeding events occurred (1.9%). In conclusion, PFS in advanced PaC patients undergoing chemotherapy is positively impacted by anticoagulation. Thromboprophylaxis with tinzaparin in treatment dose is efficient and safe.

## 1. Introduction

The prognosis for pancreatic cancer remains poor, and by 2030, pancreatic cancer will become the second-leading cause of cancer-related deaths in the United States [1]. Several studies on the use of nab-paclitaxel plus gemcitabine (N–G) used as first line treatment for advanced pancreatic cancer have emerged in recent years. The efficacy and safety of N–G was validated in the MPACT study, which showed response rates of 23% and 35% survival at one year [2]. In this study the median PFS was 5.5 months in the N–G group, compared to 3.7 months in the gemcitabine group (hazard ratio for death or disease progression, 0.69; 95% CI, 0.58 to 0.82; *p* < 0.001).

Numerous studies have reported that the risk of venous thromboembolism (VTE) in cancer patients varies widely according to primary cancer site [3]. Reported frequencies of thrombosis associated with pancreatic cancer are the highest compared to other malignancies. Pancreatic cancer has a unique ability to induce a hypercoagulable state that is associated with clinically significant thrombosis in patients, thereby conferring an increased risk of developing clots. The connection between pancreatic cancer and venous thrombosis has been discussed for almost 150 years [4]. The first case series describing the striking relationship between pancreatic cancer and thrombosis was published in 1938; it documented a 60% prevalence of venous thromboembolism in patients with pancreatic cancer at autopsy [5]. Despite the relatively low frequency of pancreatic cancer, it was reported to account for over 17% of cancer-related thromboembolism in one retrospective analysis. Studies carried out over the past 10–15 years have reported venous thromboembolism (VTE) prevalence rates of 12–36% in patients with pancreatic cancer [6]. In particular, patients with advanced pancreatic ductal adenocarcinoma, which is the most common histological type, have a seven-fold increased risk of developing arterial and venous thromboembolism compared to patients with most other solid and hematological malignancies [7,8].

We should not denigrate that the occurrence of VTE may be associated with a reduced response rate and a shorter PFS and OS among patients with unresectable pancreatic cancer. In a study of 227 pancreatic cancer patients the occurrence of a VTE during chemotherapy showed a statistically significant effect on PFS (hazard ratio (HR), 2.59; 95% CI, 1.69–3.97; *p* < 0.0001) and OS (HR 1.64; 95% CI, 1.04–2.58; *p* = 0.032). In these patients the development of VTE may have reflected the presence of a biologically more aggressive cancer that in turn led to a worse prognosis [9]. Low molecular weight heparins (LMWHs) have been used, studied, and recommended as a first-line option for the treatment and primary prophylaxis of VTE in pancreatic cancer patients [10,11,12]. Moreover, due to the very high thrombotic burden of pancreatic cancer, in the guidance of a subcommittee of the Scientific Standardization Committee (SCC) of the International Society on Thrombosis Hemostasis (ISTH), for the prevention of venous thromboembolism in cancer outpatients [13], there is a statement related to the use of treatment doses of LMWHs for prophylaxis in patients with advanced pancreatic cancer who are not otherwise considered to be at high risk for bleeding.

Additionally, many non-anticoagulant properties attributed to LMWHs have also been described. It seems that LMWHs can affect circulating tumor cells and the tumor micro-environment (TME) through various mechanisms including the effects of heparan sulfate proteoglycans/heparanase on metastasis formation, angiogenesis/tumor vasculature, and immune-suppressive/therapy-resistant TME. This ability of LMWHs to interfere with various aspects of the tumor microenvironment could, ultimately, lead to better patient outcomes [14]. There have been several experimental studies with cell lines, tumor tissue samples, and animal models in various types of cancers that have demonstrated the antitumor, anti-metastatic, and chemo-resistance reversal effect of LMWHs [15,16,17].

There is a clear need to evaluate the effects of thromboprophylaxis management beyond anticoagulation regarding the improvement of the clinical outcomes in active pancreatic cancer patients receiving systemic anti-neoplasmatic treatment. This study aimed to record PFS in pancreatic cancer patients receiving thromboprophylaxis during chemotherapy, and to compare it, using already-published data, with a reference PFS of a matched control of patients not receiving thromboprophylaxis.

## 2. Material and Methods

### 2.1. Study Design

This was multicenter, retrospective, phase IV, non-interventional cohort study that aimed to record the daily clinical practice regarding thromboprophylaxis with tinzaparin in high thrombotic risk pancreatic cancer patients undergoing chemotherapy who were administered thromboprophylaxis. Specifically, we recorded, from the four participating centers, in an outpatient setting, consecutive data from patients with advanced or metastatic pancreatic cancer under chemotherapy with N–G and receiving thromboprophylaxis with tinzaparin according to the participating centers’ clinical practice.

The primary end point was the evaluation of the PFS of patients receiving thromboprophylaxis with tinzaparin, compared with, from already-published data, the PFS of patients receiving the same chemotherapy but not thromboprophylaxis. The secondary end points was the efficacy and safety of tinzaparin in our cohort of patients. This was measured by the number of new thrombotic events observed in the study population and by the evaluation of thromboprophylaxis-related bleeding events.

Apart from objectively confirmed advanced or metastatic pancreatic cancer, other inclusion criteria were: age > 18 years, ECOG status between 0 and 2, and signed informed consent where applicable. The study complied with the Helsinki Declaration and was approved by the Bioethics Committee of all participating hospitals (Molecular Oncology Unit, Department of Biological Chemistry, Medical School, National and Kapodistrian University of Athens; Laiko General Hospital, Mitera Hospital-Hygeia Group; Ippokrateio General Hospital Athens; and Theagenion Hospital, Thessaloniki, Greece).

### 2.2. Reference PFS

The reference PFS was calculated using the PRISMA (preferred reporting items for systematic reviews and meta-analyses) guidelines for systematic reviews and meta-analysis. Eligible studies reported in PubMed, up to the study data collection time point (17 December 2020) were selected as being potentially eligible for inclusion. Only studies published in the English language were selected and there was a restriction on publication year, i.e., only publications after 1 January 2010 were requested; moreover no restrictions on publication type were imposed. The search question was formulated to include all the essential terms related to chemotherapy agents, disease, and outcome. Specifically the query question issued in PubMed for this search was: “(abraxane OR nab-paclitaxel OR albumin-bound paclitaxel) AND (gemzar OR gemcitabine) AND pancrea* AND (progression OR PFS) AND English (language) AND (“2010” (date—publication): “3000” (date—publication))”. Note the asterisk in word “pancrea*” was used to involve many terms such as pancreas (s, tic) etc.

**Figure 1:**
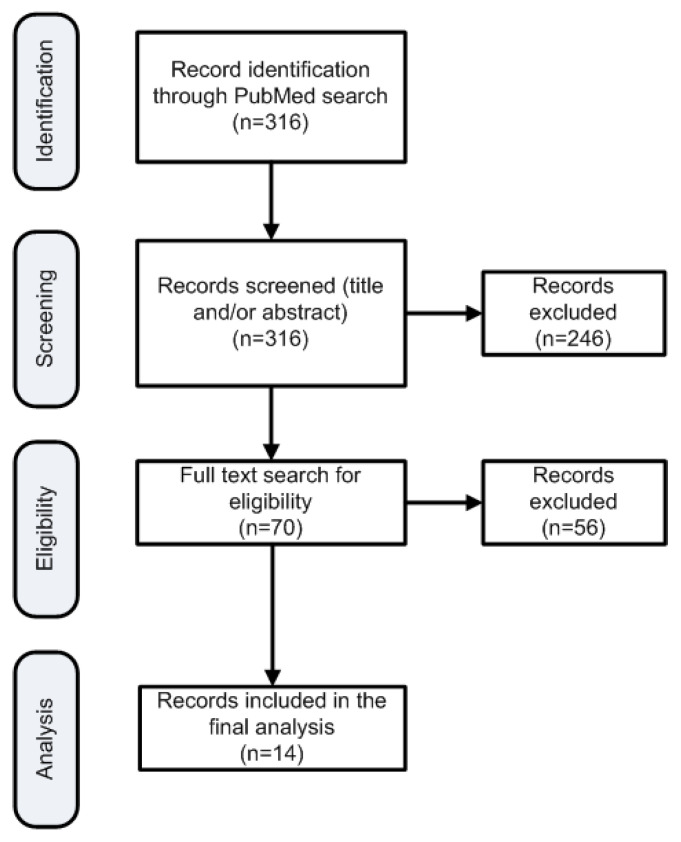
Flowchart of the search strategy to identify publications for reference PFS estimation.

This systematic search resulted in 316 publications (Figure 1). Two researchers reviewed all search results independently (screening process). The review was based on titles and abstracts. Since the PaCT study analyzed 110 patients, studies with fewer than 100 patients in the arm of N–G were excluded in order to maintain a similar or higher population. Additionally, studies that involved only locally advanced pancreatic cancer were also excluded since they had different population composition to the PaCT study. In cases of disagreement the opinion of a third researcher was requested. Other researchers participated in the data extraction stages and ensured that anticoagulation was not administered systematically in the included patients. Figure 1 depicts the various steps of data collection and selection process. Eventually, 14 publications were found eligible for inclusion in the PFS estimation. The weighted PFS number of patients in each study was used subsequently as reference point to compare with the PFS of this study for the patients systematically receiving Tinzaparin.

PFS for PaCT study patients receiving tinzaparin was defined as the time from the initiation of treatment (simultaneously with the chemotherapy) until the date of disease progression, or death from any cause, whichever occurred first.

Thromboprophylaxis efficacy was evaluated by the number of vein thromboembolic events, including pulmonary embolism and deep vein thrombosis and safety byclinically relevant non-major bleeding (CRNMB), and minor bleeding events categorized following the ISTH criteria [18]. The efficacy and safety of the anticoagulation treatment was evaluated during the period that the patients were receiving tinzaparin.

### 2.3. Statistical Analysis

Collected data from the patients were accumulated in a Microsoft Excel file (Microsoft Inc., Redmont, WA, USA). The statistical analysis was performed using the SAS^®^ version 4.0 for Windows Platform (SAS Institute Inc., Cary, NC, USA). Descriptive statistics were expressed as number and percentage for the categorical variables (such as gender and tumor location) and by the median values along with the 1st and 3rd quartiles (Q1 and Q3, respectively). Data normality could not be ensured, thus non-parametric tests were applied. Specifically, intergroup comparisons were performed using the Mann–Whitney U test. The significance level was based on *p*-values < 0.05. PFS curves were estimated by the Kaplan–Meier method and PFS differences between group were evaluated by the log-rank test.

In order to calculate the reference PFS we performed random-effects model meta-analysis using the R language (version 4.0.4) environment and the package “meta” (version 4.18-0). For the estimation of the reference studies’ homogeneity the I^2^ metric was used. In cases where the standard deviation values were missing from reference studies, these were estimated on the basis of 95% confidence intervals.

## 3. Results

### 3.1. Baseline Characteristics

Data from 110 patients fulfilling the inclusion criteria were involved in the study. About 50% of the patients were males. The patients’ median age was 68 years without significant difference in the age between males and females (median and (Q1–Q3): 67.8 (59.7–74.2) for males and 68.2 (59.9–71.8) for females, *p* = 0.9618). Similarly, no differences were found in the BMI (median BMI and (Q1–Q3): 25.0 (22.5–27.5) for males and 24.0 (22.2–26.7) for females, *p* = 0.2979). Seventy three out of 110 patients (66.4%) had not undergone surgical operation and 102 (92.7%) had metastatic disease. The majority of the tumors were grade II (69%) and fewer were grade III (18%). In 50 (45.5%) of the patients the tumor location was in the pancreas caput (head), in 29 (26.4%) it was in the corpus (body), and in 15 (13.6%) it was in the cauda (tail), while the remaining 16 (14.5%) were in mixed or other locations.

Baseline characteristics of the patient population in the PaCT cohort and in the 14 studies taken into account for comparison are depicted in Table 1 and Table 2. Characteristics depicted in Table 1 and Table 2 are comparable with the regard to the PaCT study. In more detail, the random effects age of the reference studies cumulatively was 66.6 years (I^2^ = 98%; 95% CI, 64.8–68.4 years), while for the PaCT study, the mean population age was 66.9 ± 9.4 years, which is not statistically different from the reference studies (one-sample *t*-test: *p* = 0.896). In terms of gender the PaCT study involved 51.8% men while the reference studies aggregated to 55 (I^2^ = 31%; 95 CI, 53–57%), which does not have statistical significance (comparison of proportions: *p* = 0.5078). For the tumor location, when this was located at the pancreas head, cumulatively from the studies that provided such information, the percentage of head location was 43% (I^2^ = 84%; 95% CI, 41–45%) which does not differ from the PaCT study (difference, 2.5%; 95% CI, −6.7–12.0%; *p* = 0.6025). Finally in terms of ECOG PS, in the PaCT study, all patients had PS 0 or 1 while the aggregated percentage of patients with PS 0 or 1 in the reference studies was 89% (I^2^ = 95%; 95% CI, 81–94%), which accounts for 11% difference in comparison with the PaCT study population (*p* < 0.05). Median tinzaparin dosage was 10,291 ± 1176 Anti-Xa IU, OD and median duration of administration was 8.7 (5.6–11.9) months.

### 3.2. PFS of Patients Receiving Tinzaparin

**Figure 2:**
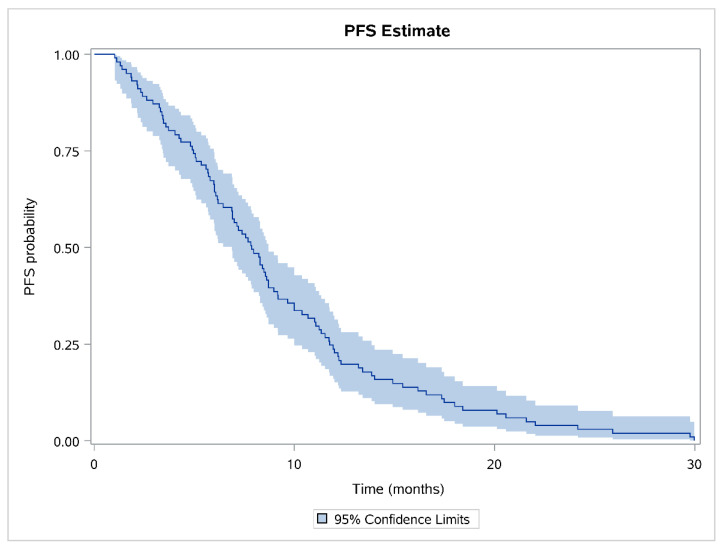
Kaplan–Meier curve for the study population.

The median PFS of patients administered thromboprophylaxis with tinzaparin was 7.85 months (95% CI, 6.87–8.71 months; Q1–Q3, 4.96–11.76; minimum, 1 month; maximum, 30 months). The Kaplan–Meier estimate along with the 95% CI is depicted in Figure 2.

### 3.3. PFS Comparison

**Figure 3:**
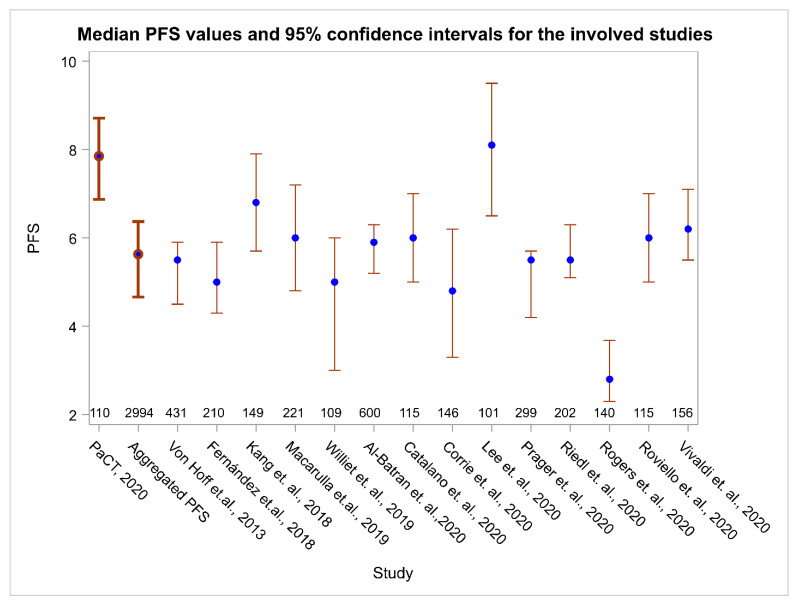
Median PFS and 95% CI for this study (the PaCT study), for the aggregated PFS (weighted PFS) and for the 14 individual reference studies. Numbers above the horizontal axis indicate the number of patients in each study.

PFS in the PaCT study cohort was 39.45% higher than the aggregated weighted PFS from all fourteen studies which was calculated to be 5.6 months (95% CI, 5.0–6.2; heterogeneity index I^2^ = 85.2%; 95% CI, 76.3–90.8%). The comparison of PFS for the patients involved in the PaCT study with PFS of patients in each individual study used as the control showed that there was significant prolongation in the median PFS (*p* < 0.05 in most comparisons) (Figure 3).

### 3.4. Study of Other Factors That Could Affect PFS

**Figure 4:**
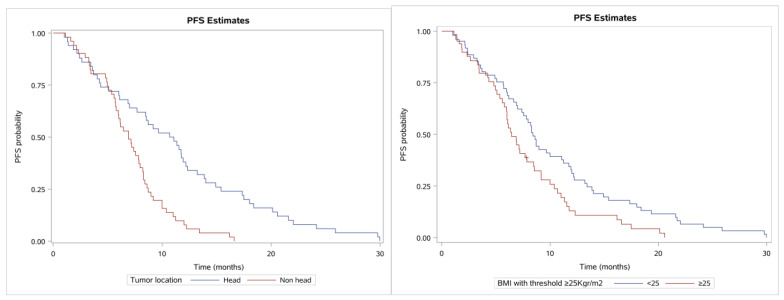
Kaplan–Meier curves showing PFS. (**left**) Patients with tumor location at the pancreas head vs. elsewhere (non-head) and (**right**) BMI with cut-off of 25 (normal vs. overweight).

Another factor affecting PFS was found to be the tumor location; specifically, patients with the tumor located only at the pancreatic head (50 patients) had higher PFS (median 10.9; 95% CI, 7.0–12.2 months; Q1–Q3 range, 4.3–15.4 months) than patients with tumor located elsewhere (51 patients) with median PFS 6.7 months (95% CI, 5.7–8.0; Q1–Q3, 4.7–8.7 months) (see Figure 4 for the related Kaplan–Meier estimates, *p* < 0.0001). Moreover, BMI with threshold 25 Kg/m^2^ also had a role in PFS as patients with BMI < 25 having a median PFS of 8.4 months (95% CI, 7.0–11.1; Q1–Q3, 5.7–13.4 months) and patients with BMI ≥ 25 having a median PFS of 6.4 months (95% CI, 5.8–7.9; Q1–Q3, 4.8–10.4 months; *p* = 0.0138). Tumor grade was not found to have a significant role in the PFS (*p* = 0.2176), PS (0 vs. 1, *p* = 0.6608), or gender (*p* = 0.9479).

### 3.5. Efficacy and Safety during Anticoagulation

In terms of anticoagulation efficacy, during the follow-up period of 18.3 ± 11.7 months, three thrombotic events were observed (i.e., 2.7%; 95% CI, 0.9–7.7%). The patients characteristics were: patient 1, female 83 years old, with BMI 22.5 and mixed tumor locations who experienced pulmonary embolism; this patient died 7.3 months after diagnosis from the disease; patient 2, female 59 years old, with BMI 36, tumor location at the pancreas body, who experienced PE and died 4.8 months after diagnosis from the disease, and patient 3, male 83 years old with BMI 21.6, tumor location at the pancreas head, who experience pulmonary embolism and died 22.5 months after diagnosis from the disease. No other thrombotic events such as portal vein thrombosis or splenic vein thrombosis were reported. In terms of safety two non-major bleeding events (1.9%; 95% CI, 0.5–7.6%) occurred—one in a male patient, 61 years old, with BMI 24, who experience a CRNM bleeding and died 43 months after diagnosis and one in a female, 70 years old, with BMI 23, who experience nose bleeding (epistaxis) and died 13 months after diagnosis.

## 4. Discussion

Pancreatic cancer is a highly aggressive type of cancer, with early extensive local invasion and rapid systemic spread. In addition to its poor prognosis and high mortality, it accounts for the highest rates of venous thromboembolic events (VTEs). The bidirectional interaction between cancer and hemostasis leads to an activation of blood cells and the coagulation system, resulting in clinically relevant thromboembolism. These processes are also suspected of enhancing cancer growth and metastatic spread [32,33]. Based on that, apart the feasibility of primary pharmacologic prevention of symptomatic VTEs in outpatients with advanced pancreatic cancer, effective thromboprophylaxis management could have positive impact in patient outcomes.

In a recent systematic review and meta-analysis of 26 studies (2056 patients) aimed at evaluating N–G as a first-line treatment for advanced pancreatic cancer patients, the median PFS ranged from 4.0 months to 8.4 months across 18 studies, and the 6-month PFS rate was 41.0% (95% CI, 30.5–51.4%) for nine studies [34]. However, in the PaCT study (2994 patients) we decided to include studies with more than 100 patients, repeat the bibliographic search in order to identify and include additional and more recent studies, and include both prospective (6 out of 14 studies) and retrospective methodologies. Our study demonstrated a significant increase of 39.5% in PFS, from 5.6 months to 7.9 months in patients receiving thromboprophylaxis with treatment dose of tinzaparin (10,000 Anti-Xa IU, OD). Apart from the use of the same chemotherapy scheme, other patient characteristics such as age, gender, PS, and metastatic stage across studies included in our analysis, were well balanced (Table 1).

The potential impact of heparins on cancer survival was first suggested in late 1970s. The benefits observed in the many of the reported studies could not be accounted for by VTE prevention alone [35]. Potential mechanisms for the effects on cancer include anti-proliferative actions of anticoagulants, anti-metastatic action via anti-angiogenesis effects, effects on cellular adhesion, epithelial–mesenchymal transition (EMT), extracellular matrix heparinase–matrix metalloproteinases, and anti-inflammatory effects on chemokine signaling and chemotaxis [35]. Recent findings indicate that inflammation plays a key role in tumor progression and survival across several cancer types [36]. Inflammation seems to be a part of a triple play along with thrombosis and cancer [37,38,39,40] and there is compelling evidence for a pathogenic role of blood coagulation in tumor growth and metastasis [41,42].

Heparin has a biological basis as a modulator of inflammation. The anti-inflammatory effects of heparin occur at multiple levels. Tinzaparin was confirmed to inhibit selectins, which have a significant role in metastasis formation, most effectively among the LMWHs [43]. In an experimental model of human colon cancer, tinzaparin administration 24 h after angiogenesis stimulation by VEGF led to a decrease of the angiogenic index to the control level. Tinzaparin exerts its anti-neoangiogenic activity as it appears to stimulate more production of tissue factor pathway inhibitor (TFPI) by epithelial cells than any other low molecular weight heparin, inhibiting tissue factor (TF) and consequently VEGFR. TFPI works by blocking the activation of protease activated receptors 2 (PAR2), the activation of which plays an important role in the metastatic potential of this type of cancer [14]. In vitro experiments have shown that the triple combination with tinzaparin, nab-paclitaxel and gemcitabine, decreases the protein levels of VEGFR2 in PC cell lines with mutant KRAS. Only the triple combination causes a decrease in *p*-ERK levels. The triple combination of tinzaparin plus N–G decreased cell viability by around 50% via apoptosis of PC cell lines harboring KRAS mutations. Furthermore, in vivo experiments in NOD/SCID mice (PANC-1 pancreatic tumor xenografts) have shown smaller tumor growth. The triple combination of tinzaparin +N–G leads to a decrease in tumor size relative to the control by 51% and relative to N–G by approximately 20%. The use of tinzaparin only reduces the tumor size compared to the control by 18% [44] in a dose-dependent way, as has been concluded from animal models.

Focusing on pancreatic cancer, the California Cancer Registry study reported the results of a multivariate analysis of potential risk factors associated with VTE within 1 year of cancer diagnosis in 6712 patients [45]. Patients with metastatic disease at the time of diagnosis had a 3.3-fold higher risk of VTE than patients with localized disease; 92.7% of patients in our cohort had metastatic disease. In a cohort of 202 consecutive patients with pancreatic cancer, Blom et al. [46] reported that individuals with a tumor of the corpus/cauda (body and tail) had a 2–3-fold increased risk of VTE compared with individuals with tumors of the caput (head) [46]. In more than half of the patients in our cohort, tumor location was corpus/cauda.

Despite the high thrombotic burden (HTB) in our cohort population thromboprophylaxis with more effective doses of tinzaparin demonstrated high efficacy in primary prevention of symptomatic VTEs in outpatients with advanced pancreatic cancer undergoing first-line chemotherapy with only three thrombotic events observed during the evaluation period. In terms of safety, during the same period there were no major bleedings; we observed only one CRNMB and one minor bleeding event. Albeit, LMHWs express pleotropic effects in a dose-dependent manner as demonstrated in pre-clinical data, the positive impact in the PFS observed retrospectively in the PaCT study clinical setting by intense (10,000–14,000 IU) anticoagulation with tinzaparin without compromising safety, supports the hypothesis that LMWHs could improve the PFS of pancreatic cancer patients.

Other studies of similar nature, such as the CONKO-004 [12] and the FRAGEM [11], had, as a secondary end point, the assessment of the role of thromboprophylaxis in patients’ PFS or survival. CONKO-004 did not find any role of thromboprophylaxis in PFS or OS, however the study was stopped when the required number of events for the primary outcome was reached and may therefore have been underpowered for the secondary study aims, as mentioned by the authors. In a similar manner, FRAGEM did not find any significant impact of thromboprophylaxis in OS (median OS was 9.7 and 8.7 months in the two arms, *p* = 0.682) and time to progression (TTP) was 5.3 and 5.5 months, respectively (*p* = 0.841). Moreover, N–G was not the anticancer treatment either in CONKO-004 or in FRAGEM. Additionally, the anticoagulation agent chosen was also different. Therefore, no further comparisons with our results were considered.

Furthermore, there are two relevant studies on the effect of thrombosis on PFS. In a single-center retrospective cohort study of 227 patients (with unresectable pancreatic cancer), the appearance of VTE during chemotherapy was found to severely affect the patients’ PFS (HR, 2.59; 95% CI, 1.69–3.97; *p* < 0.0001) [9]. In a more recent study (the BACAP trial) for pancreatic ductal adenocarcinoma of any stage, it was shown that patients who developed VTE during the follow-up period had similarly shorter PFS times (HR, 1.74; 95% CI, 1.19–2.54; *p* = 0.004) [47,48].

Our study has some shortcomings, the obvious one being that this was a retrospective series and some endpoint comparisons were based on published data; the ideal setting for such studies would be a randomized control trial. A further weakness is that we cannot account for selection bias, as we cannot identify and report in retrospect on details of all patient characteristics in reference studies included in our analysis. In addition, the preferred method to compare PFS time between the reference population and our population would be the log-rank test, however, again since no detailed data from the reference studies were available, the analysis was based on the median PFS. Moreover, the PaCT study population had PS 0 or 1 while the aggregated percentage of such patients in the reference studies was 89%. Furthermore, the aim of the study was PFS evaluation of patients receiving N–G plus tinzaparin, evaluation of survival after progression of disease was out of the study scope, and, in addition, the use of tinzaparin was not confirmed for the study participants after disease progression; moreover chemotherapy treatment was not common.

## 5. Conclusions

In conclusion, our study demonstrated a possible positive impact on PFS by intense anti-coagulation with tinzaparin in treatment doses and the high efficacy, feasibility, and safety of this approach in primary prevention of symptomatic VTEs in advanced PaC patients undergoing chemotherapy with N–G. Further prospective randomized research is needed in order to confirm the possible benefit of tinzaparin on PFS.

## Figures and Tables

**Table 1:** Baseline characteristics of the 14 studies involved for the calculation of the reference PFS and for this study (the PaCT study). *: Only patients treated with N–G. **: For these studies the combined population of two arms (both treated with N–G) was used, age was calculated as the weighted average of the two arms. ***: For this study the part of the population that continued therapy was used.

Study	Year	N *	Age (Median and Range)	Gender (Males)
Von Hoff et al. [2]	2013	431	62 (27–86)	57%
Fernández et al. [19]	2018	210	65 (37–81)	60.5%
Kang et al. [20]	2018	149	62 (36–82)	56.4%
Macarulla et al. [21] **	2019	221	69 (35–89)	52.9%
Williet et al. [22]	2019	109	70 (62–75)	49.5%
Al-Batran et al. [23]	2020	600	70 (39–86)	58.2%
Catalano et al. [24]	2020	115	65 (50–84)	53%
Corrie et al. [25] **	2020	146	65 (45–82)	56.8%
Lee et al. [26] ***	2020	101	63 (61–74)	53%
Prager et al. [27]	2020	299	70 (41–89)	56%
Riedl et al. [28]	2020	202	70 (43–89)	54%
Rogers et al. [29]	2020	140	67 (37–83)	41.4%
Roviello et al. [30]	2020	115	65 (50–84)	53%
Vivaldi et al. [31]	2020	156	71 (65–87)	53.8%
The PaCT study (this study)	2020	110	68.0 (40–86)	51.8%

**Table 2:** Clinical characteristics of the 14 studied involved in the reference PFS estimation and the PaCT study. *: For these studies the combined population of two arms (both treated with N–G) was used, PFS was calculated as the weighted average of the two arms. **: For this study the part of the population that continued treatment was used. ***: 95% CI was not reported in this study, the reported CI was estimated by using information from the other studies. PS, performance status.

Study	PFS (95% CI)	PS	Disease Stage	Location (Head)	Study Type
Von Hoff et al. [2]	5.5 (4.5–5.9)	PS0–PS1	Metastatic	44%	Prospective observational
Fernández et al. [19]	5 (4.3–5.9)	71% < PS1	Metastatic	NR	Retrospective observational multicenter
Kang et al. [20]	6.8 (5.7–7.9)	96.6% < PS0–1	Metastatic	32.2%	Retrospective
Macarulla et al. [21] *	6.0 (4.8–7.2)	PS2	Advanced or locally advanced	26.1%	Prospective observational multicenter
Williet et al. [22]	5 (3–6)	PS0–PS2	Metastatic	46.8%	Retrospective
Al-Batran et al. [23]	5.9 (5.2–6.3)	93%: PS0–PS2	Metastatic	48.7%	Retrospective multicenter
Catalano et al. [24]	6 (5–7)	PS0 or PS1	Metastatic	NR	Retrospective
Corrie et al. [25] *	4.8 (3.3–6.2)	PS0–PS2	Metastatic	47.3%	Prospective
Lee et al. [26] **	8.1 (6.5–9.5)	PS0–PS2	Metastatic	35%	Retrospective multicenter
Prager et al. [27] ***	5.5 (4.2–5.7)	94%: PS0–PS1	Metastatic and advanced	45%	Prospective observational multicenter
Riedl et al. [28]	5.5 (5.1–6.3)	>99%: PS0–PS2	Metastatic and advanced	NR	Prospective observational multicenter
Rogers et al. [29]	2.8 (2.3–3.68)	83.6: PS0–PS2	Metastatic	46.4%	Retrospective
Roviello et al. [30]	6 (5–7)	PS0–PS2	Metastatic	NR	Retrospective
Vivaldi et al. [31]	6.2 (5.5–7.1)	PS0–PS2	Metastatic	NR	Retrospective
The PaCT study (this study)	7.85 (6.87–8.71)	PS0 (72.5%), PS1 (27.5%)	92.7% metastatic	45.5%	Retrospective multicenter

## Data Availability

Restrictions apply to the availability of these data due to ethics issues.
